# Penetrating Wound of the Abdomen

**Published:** 1883-04

**Authors:** G. P. Delisser

**Affiliations:** New York City


### VII.—PENETRATING WOUND OF ABDOMEN—RECOVERY.

BY G. P. DELISSER, V.S.

Dec. 22d, 1882, I was called to see a large, bay truck horse, used in team, which, while in harness one-half hour before, had been run into and poled by another team. A sliver of wood from the broken pole had penetrated the abdominal cavity about six inches to the right of the median line and about three inches anterior to the umbilicus on the same line. There was also considerable laceration of skin and superficial abdominal fascia, around the wound, the latter being laid bare for several square inches. The wound had bled and was bleeding quite freely. On careful examination of the injury I could find no evidences of either splinter or other foreign bodies left in the skin or abdominal cavities, nor was there any hernial protrusion of the intestine. In order to explore the wound thoroughly I passed a long silver probe into the abdominal cavity at least six inches. Having become satisfied that the penetration had taken place, I washed thoroughly the wound and closed it by strong sutures, leaving a small opening in the most dependent part for the serous fluid or pus to escape. I then, by means of a piece of cotton fabric covered with Balsam of Fir, sealed up the wound, covered it with a compress and fixed it in position by means of a bandage lightly drawn around the body of the animal. I then secured him so that he could not lie down or disturb the dressing in any way. Ordered a small bran mash, and gave an opiate sufficient to secure rest, which was

Tr. opii ℥ii.

Ol. lini sem. Oi.

December 23.—Animal breathing somewhat more rapidly than normal and seemed to be suffering some pain. Thinking it might be due to the bandage I removed it and redressed essentially as before.

December 24.—Was more comfortable; breathing less frequently; seemed, however, dull and feverish; I ordered a drench of

| | |
|---|---|
| Tr. opii., | ʒii. |
| Ext. Belladonna, | ʒi. |
| Chloroformi, | ʒii. |
| Aquæ, | Oi. |

December 25, 26, 27.—Seemed quite easy. The wound kept clean. Animal fed moderately on mash and some moistened hay.

December 28.—Not so well; refused food; for first time the temperature 103, pulse 60; very uneasy; pawing quite constantly and trying to lie down; I gave hypodermic injections of morphiæ sulph., gr. vi., every hour for four consecutive hours, when rest was obtained.

December 29.—Prof. Walton of Columbia Veterinary College, saw the animal in consultation. He examined the wound, which was then beginning to suppurate and thought it was doing as well as circumstances would allow.

He advised the treatment to be continued.

December 30–31.—The animal was easier; morphiæ was given hypodermically grs. iii., t.i.d. As his uneasiness became less, the wound was washed out daily with a carbolic acid wash (1–40.)

January 1–2, 1883.—Doing better; eating mashes.

January 3.—More uneasy; breathing hurried; suffering evidently some pain; I removed bandages entirely and dressed wound with turpentine and sweet oil; gave morphine hypodermically sufficient to quiet pain grs. iii., every two hours for three times. As bowels had not moved for several days I gave an enema of warm linseed oil, which had the desired effect.

January 4.—Was a little easier; pulse 55, temperature 103, respiration, a little more rapid than normal.

January 5.—Much easier; wound freely discharging pus; washed out thoroughly with hot water carbolized.

January 6.—Broke his halter during the night, lay down, tore several sutures out and the dressing off, which produced same gaping of wound which had nearly closed in some places. As it was evident that the wound could heal thereafter only by granulations, I had the parts washed twice a day with warm water and carbolic acid (1–40) and dressed with light compresses till the wound was entirely healed, which was on January 21st, 1883.

January 28.—The horse was again put to work, being considered quite well.

New York City.

